# Maintaining Hygiene in Orthodontic Miniscrews: Patient Management and Protocols—A Literature Review

**DOI:** 10.3390/dj12070227

**Published:** 2024-07-19

**Authors:** Riccardo Favero, Martina Fabiane, Andrea Zuccon, Diego Conte, Francesco Saverio Ludovichetti

**Affiliations:** Department of Neurosciences—Dentistry Section, Università degli Studi di Padova, 35122 Padova, Italy; riccardo.favero@unipd.it (R.F.); martina.fabiane@studenti.unipd.it (M.F.); andrea.zuccon@unipd.it (A.Z.); diego.conte@studenti.unipd.it (D.C.)

**Keywords:** orthodontic miniscrews, oral hygiene, preventive dentistry

## Abstract

Background: Oral hygiene is crucial for the success of orthodontic therapy involving temporary anchoring devices like miniscrews. Plaque buildup, exacerbated by orthodontic appliances, causes inflammation that can undermine treatment outcomes. Individualized prevention plans based on patient risk factors are essential. This review emphasizes the importance of oral hygiene in orthodontic therapy with miniscrews, identifies optimal devices for ensuring long-term stability, and explores protocols for high-risk patients. Materials And Methods: A comprehensive search was conducted on two primary databases, PubMed and Google Scholar, for relevant articles on oral hygiene and inflammation. Fourteen articles meeting the inclusion criteria were selected, covering topics such as “orthodontic miniscrew”, “miniscrew and laser”, “miniscrew and mouthwash”, “electric toothbrush”, and “GBT”. Results: Inflammation can compromise miniscrew stability by damaging surrounding bone. Miniscrews of 10 mm length have lower failure rates due to better bone contact and stability. Chlorhexidine reduces inflammation risk and inhibits epithelialization around the implant head. Laser therapy enhances miniscrew stability and reduces inflammation. Chitosan effectively suppresses inflammatory mediators and prevents microorganism adhesion. Both sonic and roto-oscillating electric toothbrushes remove plaque effectively, with roto-oscillating brushes showing superior results. The Guided Biofilm Therapy (GBT) protocol offers professional hygiene benefits similar to traditional methods, with improved patient engagement and motivation. Conclusions: Home oral hygiene maintenance is paramount for preventing inflammatory complications. Professional interventions such as diode laser usage, particularly in adult patients with a history of periodontitis or underlying systemic conditions, can mitigate orthodontic therapy failure risks. The GBT protocol fosters a more comfortable and participatory professional hygiene experience for patients, promoting better oral health awareness and compliance.

## 1. Introduction

The success of orthodontic treatment hinges on effectively controlling biomechanical vectors and maintaining stable anchorage. Orthodontic miniscrews are specifically designed to streamline therapy and mitigate the limitations associated with traditional orthodontic methods and other intraoral anchorage systems. Due to their small size, miniscrews are well-tolerated by adolescent and adult patients. Their use involves standardized surgical protocols for insertion, requiring collaboration between the orthodontist and oral surgeon [1].

Orthodontic micro-implants are compact structures enabling dental movement without the need for anchoring to other elements. They differ significantly from traditional implants as they do not necessitate osseointegration, do not support prosthetics or crowns, and lack an axial load. Through the anchoring screw, the movement takes place in an orthogonal, lateral direction, deviating from its own axis [1].

Miniscrews have proven to provide reliable anchorage and have been utilized in numerous clinical applications such as correcting deep bites, closing spaces, correcting midlines, extrusion, intrusion, distalization, mesialization, and mass retraction, with a high success rate. The use of miniscrews has expanded the scope of non-surgical orthodontic therapy [2].

The stability of orthodontic mini-screws is influenced by several critical factors, including the quality and quantity of bone at the insertion site, which are essential for achieving secure anchorage. The experience and precision of the orthodontist play a significant role in correctly placing the mini-screw and selecting the appropriate size and type for the specific anatomical site. Additionally, the design and material of the mini-screw affect its ability to integrate and maintain stability within the bone. Patient-specific factors, such as general health and lifestyle choices like smoking, can impact the healing process and the long-term success of the mini-screw. Moreover, the technique used during insertion, whether it involves pre-drilling or self-tapping screws, also affects the stability. Proper oral hygiene is essential to minimize complications related to orthodontic miniscrews. It is a key factor for the success of the therapy, ensuring hygienic maintenance during follow-up [3].

Among the preferred sites for inserting orthodontic anchorage miniscrews are the interradicular spaces on the vestibular side. However, this positioning carries risks of failure, screw fracture, or damage to the dental root, along with the challenge of assessing available bone. Therefore, it is crucial to conduct a careful analysis of three key factors: the biomechanics of the implant device, the patient’s anatomy, and the dimensions of the screw [4].

A 2011 study by the Department of Orthodontics at the University of Homburg, Germany [5], analyzed virtual models to identify optimal spaces for miniscrew insertion. Key findings showed that the best placement areas are between the maxillary central incisors, between the lateral incisor and canine, and between the second premolar and first molar. In the mandible, suitable spaces extend from the canine to the molars, with the anterior palate also identified as an optimal area for insertion.

To minimize the risks associated with the application of miniscrews, it is essential to carefully assess and select patients who meet the necessary requirements for using this device [6,7,8,9,10].

The risks associated with the use of miniscrews should be well-known to both the clinician and the patient. Complications can arise both during their placement and after orthodontic loading. The most frequent complications of a treatment involving miniscrews include fracture, which may occur during placement or removal, as well as mobility and inflammation of the soft tissues at the insertion site [11,12].

The risks and complications associated with the use of miniscrews decrease or may not occur when the insertion procedures and loading times are adhered to, excellent oral hygiene is maintained, and the clinician has good experience in handling the necessary insertion tools [2,3,13,14].

Injuries that exclusively involve the external surface of the dental root, without pulp involvement, often do not impact the prognosis of the tooth and undergo spontaneous healing with the deposition of reparative cement in all areas that come into contact with the mini-screw [3].

The miniscrews achieve primary stability through mechanical retention and can move within the bone. This movement follows the direction of orthodontic force and is not dependent on the insertion angle or length of the miniscrew.

Additionally, the experience and skill of the practitioners play a crucial role in the success rate of the miniscrew [2].

Minor aphthous ulcers are typically caused by trauma to soft tissues but can also result from genetic predisposition, bacterial infection, allergy, hormonal, vitamin, or immunological issues, or in some cases, psychological factors. Minor aphthae resolve within 7–10 days without scarring.

Inflammation and peri-implantitis can arise following the application of miniscrews, especially if the tissues are mistreated or undergo torsion during insertion. Employing an atraumatic process during miniscrew insertion reduces complications and stress on the tissues.

Peri-screw inflammation has been associated with miniscrew failure. In patients with poor oral hygiene, inflammation may occur even if the placement procedure is performed with care [2].

Maintaining home hygiene is crucial for the success of orthodontic miniscrews, as it is a daily routine that patients can manage themselves. Effective oral care, including regular brushing and flossing, helps prevent inflammation and complications around the screw sites. By adhering to these hygiene practices daily, patients play a significant role in ensuring the longevity and effectiveness of their orthodontic treatment [15].

Infection control is a fundamental factor to ensure the stability of the miniscrew. Local disinfectants, antiseptic mouthwash, and accurate brushing techniques are recommended. In more severe cases, antibiotics or the removal and repositioning of miniscrews in another site may be necessary [2]. 

Osteointegration surrounding the miniscrew is not highly desirable as it would complicate the removal by increasing torque removal values [16].

An advantage offered by the insertion of miniscrews during orthodontic treatment is the reduction in discomfort that the patient might experience if extraoral anchorage techniques were used. In general, orthodontic removal of the miniscrew is not considered a traumatic approach. However, after removal, there will be a temporary full-thickness defect through the soft tissues and the underlying alveolar bone, which undergoes secondary intention healing [6].

The aim of this review is to understand the role of oral hygiene in orthodontic treatment with miniscrews, identify the most effective measures to ensure lasting stability, and investigate the possibility of creating specific protocols for patients at risk of treatment failure.

## 2. Materials and Methods

Review Question:

The question of research was as follows: what is the role of oral hygiene in orthodontic treatment with miniscrews?

Inclusion Criteria:

- Studies that focus on oral hygiene protocols in the context of orthodontic treatment with miniscrews.
- Studies published in English.
- Study types included randomized clinical trials, literature reviews, and cohort studies.

Exclusion Criteria:

- Studies not specifically addressing oral hygiene protocols in the context of miniscrews.
- Papers not written in English.
- Studies outside the scope of randomized trials, literature reviews, and cohort studies.

Search strategies and data collection:

The reference database used for this review was PubMed. A comprehensive literature search was carried out for the key- words “orthodontic”, “mouthwash”, “miniscrew” “laser”, “electric toothbrush”, “GBT” using the Boolean operator “and” and “or” to combine these words. Only papers in English were considered. 

Independent title and abstract reviews were carried out by the authors for the purpose of including or cutting out potentially appropriate articles. Furthermore, independent full article reviews were completed on the articles considered from the initial search to assess their relevance to the research question. 

Study Selection and Analysis:

The selection process involved a two-stage screening: an initial review of titles and abstracts, followed by a detailed review of the full texts of potentially relevant articles. Studies meeting the inclusion criteria were analyzed based on their methodologies, outcomes, and relevance to the role of oral hygiene in orthodontic treatment with miniscrews. Data extraction focused on key variables related to inflammation, miniscrew stability, and the effectiveness of different oral hygiene protocols.

## 3. Results

Using the search strategy, a total of 65 articles were identified. Out of these, only 14 met the inclusion criteria and were selected for inclusion in the present review. 

Table 1 provides an overview of selected studies addressing the topic of “orthodontic miniscrews”. Subsequently, Table 2 elucidates studies that establish a correlation between “miniscrews” and “laser” interventions. Table 3 delineates selected studies that investigate the relationship between “miniscrews” and the application of “mouthwash”. Finally, Table 4 encapsulates studies examining the intersectionality of “electric toothbrush”, “GBT” (Guided Biofilm Therapy), and “orthodontic” modalities. Fourteen studies and their corresponding literature review articles were included. Of these, 12 were randomized clinical trials, 1 was a literature review, and 1 a cohort study. Table 1 clusters and briefly unfolds all of them. 

The studies have investigated the presence of specific protocols of oral hygiene in orthodontic treatment with miniscrews.

The analyzed studies had experiment periods that varied but were generally within a few months.

The fundamental requirement for patient inclusion in almost all selected studies is excellent oral hygiene. Only in study III is there a division of the sample into three groups based on the level of hygiene: good, fair, and poor.

Periodontally compromised patients were mentioned by Coronel-Zubiate in 2020 [14], where GBT treatment is identified as an adjunct in biofilm removal and periodontal maintenance, even in patients undergoing orthodontic therapy.

In study I, it was observed that despite the good hygiene of the patients, inflammation still developed in some cases. If the affected miniscrews were 10 mm long, inflammation did not cause a loss of stability, as opposed to what occurred in shorter ones. In study II, it was also demonstrated that longer miniscrews had a better long-term prognosis. 

Several factors can compromise the stability of miniscrews, including patient oral hygiene, coexisting diseases, and smoking.

In study III, it was demonstrated that patients with poor oral hygiene achieved less success. Miniscrews inserted into attached gingiva had greater success than those inserted into mobile gingiva.

In study IV, the levels of IL-1β decreased significantly between T0 and T3. The diode laser was administered to patients at four different times: T0 during miniscrew placement, T1 after one week, T2 after one month, and T3 after three months. According to this study, the use of the diode laser might be recommended after miniscrew placement.

Study V highlights that laser therapy can be recommended as a clinical adjunct to increase the success of miniscrews. However, being a study with a short timeframe, there is a need for additional samples to demonstrate that the laser is truly effective in ensuring the stability and success of miniscrews [31].

From study VI, it was observed that laser therapy can improve the stability of orthodontic miniscrews but without significant results. Laser therapy has a clear influence on the inflammatory reduction, as detected in the laser group compared to the control group.

From study VII, it emerged that patients administered with chitosan showed a lower level of iNOS and COX-2 expression [32,33].

The chitosan-based mouthwash has proven to be an effective suppressor of inflammatory mediators. As an antibacterial agent in the form of a rinse, it has been used to eliminate the adherence of oral microorganisms to the surface of miniscrews.

In study VIII, the use of fluoride mouthwashes was found to potentially cause corrosion in the form of cracks and/or pits on the surface of miniscrews after 28 days of immersion. CHX was toxic to gingival fibroblasts, and the level of toxicity depended on the concentration and exposure time. This increased toxicity is likely due to the toxic effect of releasing metal ions bound to titanium miniscrews in mouthwashes, causing a decrease in the strength of the miniscrews. Miniscrews inserted in samples of chitosan-based mouthwash and distilled water did not show significant toxicity to gingival fibroblasts.

In study XI, a statistically significant decrease in microorganisms was detected in the groups administered mouthwashes with CHX, essential oils, and 7.5% povidone-iodine, compared to the control group that did not use mouthwashes.

From study X, it was noted that the chitosan-based mouthwash has antibacterial activity similar to CHX but is more effective against red-complex bacteria [34].

From study XI, it emerged that electric toothbrushes are effective in plaque removal, but with the rotary-oscillating type, the decrease was significantly greater compared to the sonic type.

From the results of study XII, we can say that for pediatric patients, there is strong evidence that the electric toothbrush is the most effective means of biofilm removal, both for children undergoing orthodontic treatments and those who are not.

Study XIII demonstrates that applying mild orthodontic force in combination with vibratory stimuli leads to increased IL-1β secretion and an acceleration of orthodontic movement. The greater amount of movement observed in the experimental site could be attributed to the effects of increased IL-1β secretion because of vibratory stimuli.

Study XIV discusses a new method that is useful for removing biofilm around the tooth and implant structures, with clinical results better or comparable to SRP (scaling and root planing). These results have been confirmed with a reduction in microbial load and a decrease in inflammatory cytokines. Patient motivation is essential for effective treatment results. To completely remove biofilm as gently as possible, the use of a very fine powder, erythritol (14 microns, average particle size), is beneficial. It has a pleasant taste as a non-cariogenic sugar, is suitable for any type of patient, does not affect glycemic or insulin balance, has no caloric potential, and is inert, soluble, and extremely safe. 

It is activated and can be applied to dental, root, implant, or resinous or ceramic prosthetic material surfaces, as well as on the gums and back of the tongue, without causing damage, working at controlled pressures.

Below are Table 5, Table 6 and Table 7 summarizing protocols based on the selected articles for this review, applicable to the three categories of patients undergoing orthodontic treatment with miniscrews.

## 4. Discussion

Patients undergoing orthodontic treatment with miniscrews may fall into different age groups, including children, adolescents, or adults. Each category presents distinct characteristics, necessitating diverse approaches from dental professionals in the clinic.

Various reasons prompt a child to undergo orthodontic treatment, typically revolving around malocclusion or functional concerns. The child’s experience is influenced by the dental environment, the dentist, the assistant, the hygienist, and the quality of interaction with these figures. Creating an environment where the child feels welcomed, valued, and loved fosters trust and acceptance of all necessary therapies.

It is crucial to recognize that a child under the age of 8, without adequate preparation by the parent or a positive reception by the dentist, perceives the dental session as a form of violence. Therefore, respecting each patient’s time and listening attentively are essential [35].

Once the trust of the young patient is established, the dental session becomes straightforward and enjoyable, facilitating their involvement in homecare routines, with the parent taking an active role.

The role of the parent is pivotal in this age group. The child should not bear the burden of the adult’s anxiety but should be reassured and encouraged. The parent must understand the significance of the therapy for their child and actively participate in homecare maintenance. Providing education to the parent first and then to the young patient is essential.

The child typically arrives without traumatic experiences, and by employing effective approaches, it is possible to instill trust in a future teenager and adult [36].

In contrast to children, adolescents have a desire to understand the causes of phenomena and behaviors. They consistently challenge all the messages (rules, family myths, habits, ideals, etc.) conveyed by family and school to determine their genuine usefulness and alignment with their needs [37].

An adult can be a patient who has already undergone orthodontic treatment in the past. The professional may have a young patient seeking orthodontics for aesthetic reasons or an older patient who requires orthodontic treatment to address issues resulting from periodontal disease [24].

The psychological aspect in an adult patient undergoing orthodontic treatments can be the result of an entire life of both positive and negative experiences. Often, the negative experiences prevail, and the patient, aware of their past, may be hesitant to improve their current situation due to fear of the dentist, the duration of the therapy, uncertain results, and their inconsistency in following the treatment plan [38]. 

A younger patient is generally more motivated to achieve an aesthetic appearance in line with societal beauty standards and is often willing to make compromises to attain the desired outcome.

The more mature patient does not consider the external appearance as fundamental and is more motivated to achieve oral and functional well-being, both for chewing and for socializing without worrying about the opinions of others. The professional must understand the real reasons why an adult patient decides to undergo orthodontic treatment, advising them appropriately and evaluating together all the possibilities to determine whether the desired results can be achieved. It is essential to be comprehensive in explaining the phases the patient will go through, along with all the homecare practices they need to adopt to ensure the success of the treatment [39].

Improving one’s smile, enhancing personal appearance, and addressing functional issues are the primary motivations prompting patients of all ages to undergo orthodontic treatment. Understanding the psychological aspects of patients is also critical. For instance, addressing anxiety and fear in adult patients through effective communication and reassurance can significantly improve compliance and treatment outcomes. The role of psychological support in orthodontic therapy should not be underestimated and warrants further exploration. It is crucial to consider not only the aesthetic and functional aspects but also how orthodontic treatment can enhance overall satisfaction in life. Research by Barrera-Chaparro 2023 [40] suggests that incorporating satisfaction with life into discussions about oral health habits and orthodontic treatment needs can provide valuable insights into patient motivation and treatment outcomes.

Oral hygiene has been identified as the key to the success of orthodontic therapy. Therefore, it becomes crucial for patients to be aware of the necessary home care practices before the start of any orthodontic treatment. Orthodontic treatment can impact the periodontium, leading to gingivitis and gingival recessions.

The effectiveness of oral hygiene during orthodontic therapy is deeply influenced by factors such as age, socioeconomic status, pain, and patient knowledge. Younger patients, for example, typically need more guidance and may not be as consistent with their hygiene practices as adults. Socioeconomic status also plays a crucial role; individuals with higher socioeconomic backgrounds usually have better access to healthcare resources, which can lead to better hygiene practices and outcomes. Conversely, those from lower socioeconomic backgrounds might face more challenges in accessing care, leading to poorer oral health. Additionally, the pain associated with orthodontic devices can discourage patients from adhering to proper cleaning routines, increasing the risk of plaque accumulation. Lastly, a lack of knowledge about effective oral hygiene techniques can prevent patients from taking adequate care of their orthodontic appliances and oral health [41].

On the other hand, it is important to consider the impact of different orthodontic modalities on oral hygiene practices. Aligner therapy, for instance, offers certain advantages over fixed appliances in terms of oral hygiene. With aligners, patients can remove the appliances during meals and oral hygiene routines, allowing for easier cleaning of teeth and appliances compared to fixed braces. The ability to remove aligners facilitates access to all dental surfaces, enabling more thorough and effective cleaning. Long-term follow-up studies are needed to evaluate the sustained impact of different oral hygiene protocols on the stability and success of orthodontic treatments with miniscrews. These studies should assess not only clinical outcomes but also patient satisfaction and quality of life post treatment. Moreover, taking out aligners for cleaning allows patients to more easily use dental floss and interdental brushes, aiding in better plaque removal between teeth and reducing the risk of cavities and gum diseases. Research suggests that incorporating aligner therapy into orthodontic treatment plans can lead to an increase in oral hygiene possibilities and potentially improve treatment outcomes.

Orthodontic appliances can act as plaque retention devices, compromising plaque control. It is essential to consider the challenges that may arise during orthodontic treatment, such as the differences between power chains and coils used for mini-implants. Power chains, while effective in applying continuous forces to move teeth, can pose challenges for oral hygiene due to their design. In contrast, coils used for mini-implants may offer better hygiene opportunities. By addressing these differences and emphasizing the importance of maintaining proper oral hygiene practices, clinicians can help mitigate potential complications and improve treatment outcomes.

We can say that the broader complications of gingivitis on a healthy periodontium (children and adolescents) are mostly limited; however, gingivitis during orthodontic treatment can cause periodontal destruction in adults with active periodontitis. Poor oral hygiene should be a relevant motivation to discourage orthodontic therapy or determine its discontinuation, preventing the onset of more significant issues related to biofilm accumulation that could make the situation irreparable. Treatment timelines are improved when undesired effects are reduced, and maximum patient cooperation is achieved [42].

Unlike an endosseous dental implant that osseointegrates, miniscrews achieve primary stability through mechanical retention and can move within the bone following the direction of orthodontic force. Once the therapy is completed, they are removed using the same instruments used for insertion, and the area heals quickly.

To prevent ulceration, improve patient comfort, and avoid the onset of inflammation in soft tissues, a healing abutment with a wax ball placed over the miniscrew head, combined with the administration of 0.12% chlorhexidine, is recommended. 

To prevent the formation of a fibrous capsule around the screw head (a plaque receptacle hosting Gram-negative bacteria, leading to inflammation and peri-implantitis), it is advisable to position the miniscrew in keratinized mucosa or use longer miniscrews, leaving 2–3 spirals exposed to facilitate hygiene [43].

Finally, a general overview about Green dentistry must be conducted as it aims to reduce environmental impact by using biocompatible materials and minimally invasive techniques. The use of natural polymers, such as chitosan, is gaining popularity due to their antibacterial and anti-inflammatory properties. In orthodontics, chitosan coatings on appliances and miniscrews can improve hygiene and reduce inflammation. These innovations not only enhance oral health outcomes but also promote environmental sustainability. By incorporating green practices and natural polymers, dental professionals can provide more effective and eco-friendly treatments [44].

Several limitations must be acknowledged in this review. One significant limitation is the potential publication bias due to the selection of articles from PubMed and Google Scholar, which may not cover all relevant studies, especially those published in non-English languages or less accessible databases. Additionally, the variability in study designs and sample sizes among the included studies could affect the generalizability of the results. In discussing the findings of this review, it is important to acknowledge both its strengths and limitations. One of the strengths lies in the comprehensive examination of orthodontic treatment across different age groups, including children, adolescents, and adults. By delineating the unique characteristics and challenges faced by each age cohort, this review provides valuable insights for dental professionals in tailoring treatment approaches to meet individual needs. Additionally, the incorporation of diverse perspectives on patient experiences and motivations enhances the richness of the discussion.

However, it is essential to recognize certain limitations inherent in the review process. Firstly, the reliance on the existing literature may introduce bias, as the selection of articles is subject to the availability and quality of the published research. Furthermore, the scope of the review may not encompass every aspect of orthodontic therapy with miniscrews, potentially overlooking emerging trends or alternative treatment modalities. Future research efforts should aim to address these limitations by adopting more rigorous methodologies and expanding the scope of investigation. Further research is also needed to explore the long-term effects of different oral hygiene protocols on miniscrew stability across diverse patient populations. Investigating the impact of socioeconomic factors on oral hygiene practices during orthodontic treatment can provide deeper insights into addressing disparities in treatment outcomes. Moreover, the development and evaluation of new biocompatible materials, such as chitosan, in orthodontics warrant continued investigation to enhance patient care and sustainability in dental practices.

## 5. Conclusions

The accumulation of plaque and biofilm around miniscrews significantly increases the risk of inflammation and stability loss. Placing miniscrews in keratinized mucosa aids in daily cleaning and inflammation prevention. Chitosan-based mouthwash is effective against periodontal bacteria, especially in high-risk patients. Improving patient engagement and motivation is crucial for better home care routines, with dental floss and electric toothbrushes being useful tools. Professional interventions like diode lasers and the GBT protocol help prevent inflammation and remove plaque. Future research should focus on long-term studies to assess the efficacy of hygiene protocols.

## Figures and Tables

**Table 1:** Orthodontic miniscrew studies included in the literature review.

Orthodontic Miniscrew					
	Reference	Country	Methodology	Aims/Purpose	Key Findings and Results
I	Michał Sarul et al. (2022) [17]	Poland	RTC	A total of 184 miniscrews, with lengths 8 mm and 10 mm, were analyzed. There were 92 patients with excellent oral hygiene.	Success rate with inflammation is 64.29%. Success rate without inflammation is 94.74%. The 10 mm miniscrews, even though they had more inflammation, were more established.
II	Michał Sarul et al. (2015) [18]	Poland	RTC	A group of healthy patients with excellent oral hygiene were fitted with miniscrews with lengths 6 mm and 8 mm.	Numerous factors can compromise the stability of miniscrews including the patient’s oral hygiene, coexisting diseases, and smoking. Longer miniscrews had better stability over time.
III	P. Sharma et al. (2011) [19]	India	RTC	A total of 139 miniscrews with length 8 mm. They were placed in 73 patients. The sample was divided into 3 groups: good, fair, or poor.	Patients with poor IOD and a high mandibular angle were seen to have less success. Implants placed in the keratinized gingiva were more successful than those inserted in the mobile gingiva.

**Table 2:** Miniscrews and laser studies included in the literature review.

Miniscrew and Laser					
	Reference	Country	Methodology	Aims/Purpose	Key Findings and Results
IV	Soghra Yassaei et al. (2023) [20]	Iran	RTC	Eighteen patients had miniscrews placed between the premolar and the upper first molar. The diode laser was used at a wavelength of 980 nm and at a power of 100 mw in continuous wave mode.	IL-1ß levels decreased significantly from application and after 3 months. The use of the diode laser could be recommended after the placement of the miniscrew.
V	Özer Alkan et al. (2021) [21]	Turkey	RTC	In 15 people, the miniscrews were inserted in the interadicular region between premolar and maxillary first molar.	Study highlights laser therapy may be recommended as a clinical adjuvant to increase the success of the miniscrews.
VI	Aly Osman et al. (2017) [22]	Egypt	RTC	Twelve patients with 34 miniscrews. This study evaluated the stability of immediate-loading miniscrews in the buccal alveolar bone of the maxilla and gingival inflammation peri-implant after laser therapy application.	LLLT improved the stability of orthodontic miniscrews, and had a definite influence on reducing gingival inflammation around miniscrews.

**Table 3:** Miniscrews and mouthwash studies included in the literature review.

Miniscrew and Mouthwash					
	Reference	Country	Methodology	Aim/Purpose	Key Findings and Results
VII	Haru Setyo Anggani et al. (2021) [23]	Indonesia	RCT	Fifty-three minivites in 30 subjects divided into 3 groups: the 1st received 1% chitosan mouthwash, the 2nd 0.2% chlorhexidine digluconate, and the control group received distilled water.	Patients who were given chitosan had a lower level of iNos and COX-2 expression.
VIII	Wulan S. Utami et al. (2022) [24]	Indonesia	VS	Twenty-eight miniscrews immersed in 4 different mouthwash groups: CHX 0.2%, sodium fluorinated mouthwash 0.2%, chitosan mouthwash 1.5% and distilled water.	Chitosan and distilled water mouthwash showed no fibroblast toxicity, unlike CHX and sodium fluodate.
IX	Y Akbulut. (2020) [25]	Turkey	RCT	Thirty-eight patients divided into 4 groups, each with 15 miniscrews.	Significant decrease in microorganisms in the CHX, essential oils, and povidone-iodine groups compared to the control group.
X	Erlina Hasriati et al. (2020) [26]	Indonesia	RCT	Thirty patients randomly assigned to chitosan rinse (10), CHX (10), and the last 10 with distilled water.	Chitosan-based mouthwash is better on red-complex bacteria.

**Table 4:** Electric toothbrush, GBT (Guided Biofilm Therapy), and orthodontic studies included in the literature review.

Electric Toothbrush and Orthodontic					
	Reference	Country	Methodology	Aim/Purpose	Key Findings and Results
XI	Christina Erbe et al. (2019) [27]	Germany	RCT	Oscillating–rotating electric toothbrush with orthodontic head was compared with sonic toothbrush, in 44 orthodontic patient > 12 years.	Plaque reduction was significantly greater with the roto-oscillating toothbrush than with the sonic.
XII	Andrew Graves et al. (2023) [28]	USA	SR	The inclusion criteria: pediatric patients who used manual or electric toothbrushes. Exclusion criteria: non-human subjects, studies involving only adult patients, non-dental clinical applications.	High reduction in plaque index scores among pediatric patients using electric toothbrushes.
XIII	Chidchanok Leethanakul et al. (2016) [29]	Thailand	RCT	Fifteen patients in ortho therapy for canine distalization.	Electric toothbrush improved IL-1β secretion in GCF and accelerated orthodontic tooth movement.
**GBT and Orthodontic**					
	**Reference**	**Country**	**Methodology**	**Aim/purpose**	**Key findings and results**
XIV	Deepti Shrivastava et al. (2021) [30]	Arabia Saudita	SR	This review explores the various aspects of GBT along with its substantial use in the treatment of periodontal and peri-implant diseases.	GBT in periodontal and peri-implant disease is very useful and is an effective means of removing biofilm from the tooth and around an implant.

**Table 5:** Protocols based on miniscrew and pediatric patients.

Miniscrew Pediatric Patient				
	Laser	Mouthwash	GBT	Electric Toothbrush
Professional Hygiene	NOT recommended in healthy patient without periodontal disease.		GBT is painless, motivates children, and helps prevent tooth decay.	
Home Hygiene		Mouthwashes were tested on 18-year-old plus patients.		Strong recommendation of a roto-oscillating toothbrush, with the aid of dental floss and/or small interdental brushes. A single tuft toothbrush is useful to clean around the miniscrew. It is also very useful using the plaque detector after brushing. *

* In the pediatric patient, it is essential that the adult who supervises him/her is adequately instructed and helps the child with home oral hygiene maneuvers, until the attainment of complete autonomy.

**Table 6:** Protocols based on miniscrew and adolescent patients.

Miniscrew Adolescent Patient				
	Laser	Mouthwash	GBT	Electric Toothbrush
Professional Hygiene	It may be recommended for situations of severe inflammation (gingivitis, mucositis) in patients at risk with pathologies.		Strongly recommended for the motivation given to the young patient	
Home Hygiene		Chitosan mouthwash is recommended. It can be used soaked in dental floss, interdental brush, or single tuft toothbrush to better clean the spaces between the teeth and around the miniscrews.		A roto-oscillating toothbrush with the help of dental floss and interdental brushes is strongly recommended. The plaque detector is also useful.

**Table 7:** Protocols based on miniscrew and adult patients.

Miniscrew Adult Patient				
	Laser	Mouthwash	GBT	Electric Toothbrush
Professional Hygiene	Valid for periodontal patients, with pathologies such as diabetes. The diode laser increases collagen synthesis and fibroblast proliferation, promotes the healing process, decreases inflammatory reactions and pain.		Strongly recommended for patient motivation and comfort in professional hygiene sessions.	
Home Hygiene		Chitosan mouthwash is recommended. It is better than CHX on red-complex bacteria in periodontal patients.		A roto-oscillating toothbrush with soft mode around the miniscrew + dental floss, interdental brush, and single tuft toothbrush soaked in mouthwash is strongly recommended.
